# Circulating lipidome underpins gender differences in the pathogenesis of type 2 diabetes

**DOI:** 10.1016/j.jlr.2025.100816

**Published:** 2025-04-26

**Authors:** Madhusmita Rout, Oliver Fiehn, Dharambir K. Sanghera

**Affiliations:** 1 Department of Pediatrics, College of Medicine, University of Oklahoma Health Sciences Center, Oklahoma City, OK; 2 UC Davis West Coast Metabolomics Center, Davis, CA; 3 Harold Hamm Diabetes Center, University of Oklahoma Health Sciences Center, Oklahoma City, OK; 4 Department of Pharmaceutical Sciences, University of Oklahoma Health Sciences Center, Oklahoma City, OK; 5 Department of Physiology, College of Medicine, University of Oklahoma Health Sciences Center, Oklahoma City, OK; 6 Oklahoma Center for Neuroscience, University of Oklahoma Health Sciences Center, Oklahoma City, OK

**Keywords:** Asian Indians, clinical lipidomics, gender, obesity, T2D

## Abstract

Metabolic alterations in human lipidome significantly impact various chronic diseases including type 2 diabetes (T2D). However, epidemiology and clinical studies have yet to identify clinically meaningful lipid markers for T2D. Fatty acids (FAs) are the backbone of lipid species. However, conflicting results on the essential FAs including omega 3 and omega 6 in the development of metabolic diseases urge deeper evaluations of diverse clinical cohorts including underrepresented populations. This study investigated the lipidomics profiles of 3,000 individuals from a well-characterized cohort of Asian Indians. Untargeted lipidomic profiles were created using blood samples applying reversed-phase liquid chromatography-accurate mass tandem mass spectrometry. Free FAs and lysophosphatidylcholines (LPCs) were upregulated, while sphingomyelin and phosphatidylcholines were decreased in T2D. We observed a significant increase of essential FAs—FA20:4 (AA), FA20:5 (EPA), and FA22:6 (DHA) in T2D after adjusting for age, gender, and body mass index. However, most ω-3 and ω-6 FAs were reduced by 2 to 6-fold in obesity in both genders. We also observed gender differences in age-associated lipid patterns in which cholesterol sulfate and LPC 22:6 were elevated in all age groups in men, but LPC 22:6 rapidly increased after menopause in women, and sphingomyelins increased in men after 40 years. Machine learning analysis identified long-chain FAs, ether-based LPCs, and clinical risk scores among the most informative features associated with T2D. Our study identified lipidomic markers that could be potential mediators of T2D and obesity. Their patterns may underpin gender differences in the pathogenesis of metabolic and cardiovascular diseases.

Type 2 diabetes (T2D) is a major disorder of metabolism that affects all global communities disproportionally. South Asians have a higher propensity to develop T2D than many other ethnic and racial populations, with up to six-fold higher risk for T2D than Europeans (1, 2). There has been a steep rise in T2D cases among South Asians due to urbanization and population growth (3, 4). In South Asians, T2D occurs about 10–12 years earlier and at lower body mass index (BMI) thresholds (5, 6). However, the underlying causes of this disparity are currently unknown and are not explained by conventional risk factors of T2D or by the currently available findings from genetic studies (7). Additional tools are necessary to identify molecular biomarkers to understand the pathophysiology of T2D, its heterogeneity, and disparities.

Lipids play a crucial role in metabolism by influencing cell signaling, energy storage, and cell membrane composition and permeability. Metabolic alterations of lipid species significantly and adversely impact various diseases, such as T2D, heart disease, neurodegenerative diseases, and cancers (8, 9, 10). The human lipidome comprises hundreds to thousands of molecular lipid species that are structurally and functionally diverse (11, 12). Lipid subclasses, including free fatty acids (FAs), ceramides (Cers), sphingolipids, phospholipids, and triacylglycerols (TGs), are known to be differentially regulated in T2D (13, 14). The impact of FAs on T2D and their dietary sources across various food groups has garnered considerable attention in research over the last 20 years. This focus stems from conflicting findings in numerous cohort and observational studies, particularly concerning the essential FAs, including omega-3 (n-3) and omega-6 (n-6) monounsaturated FA (MUFA) and polyunsaturated FA (PUFA). While several studies indicate that n-3 and n-6 MUFA and PUFA may offer protective benefits against T2D, other studies report contrasting effects. This discrepancy highlights the complexity of dietary FAs and their derivatives and their influence on metabolic health. More studies in ethnically and culturally different cohorts are needed to identify putative metabolite phenotypes that can be clinically used for disease presentation, prediction, or prevention.

LC-MS/MS based profiling of untargeted lipidomics can provide a comprehensive depiction of the human lipidome in serum and plasma (15, 16). Gender-specific regulation of cholesterol and other known lipids, lysophospholipids, and phosphatidylcholine (PC) have been reported to confer distinct profiles concerning obesity and longevity in men and women (17, 18). South Asian men have a significantly higher mortality rate due to cardiovascular disease (CVD) than South Asian women (19). However, the molecular mechanisms underlying these differences have not been explored. In the present study, we investigated untargeted lipidomics profiles of 3,000 Asian Indian participants to identify their association with T2D, particularly the role of essential FAs and their derivatives in T2D and CVD risk factors. In this study, we identified differentially regulated lipids in T2D and obesity. Our findings further revealed that the association between certain classes of metabolites and T2D or BMI was dependent on gender. Utilizing machine learning approaches, we developed prognostic models and identified high-risk individuals by integrating lipidomic data and clinical risk scores (CRS).

## Materials and Methods

### Study cohort

A total of 3,000 individuals (1725 T2D cases and 1,275 controls) were included in this study from the Asian Indian Diabetic Heart Study (AIDHS)/Sikh Diabetes Study (SDS) (20, 21, 22). The Sikh population is a relatively homogenous endogamous community from India. Sikhs are primarily nonsmokers, and ∼ 50% of them are vegetarians. However, the incidence of T2D and CVD in Sikhs and South Asians has markedly increased over the past two decades (7, 23). T2D was diagnosed based on their medical records for symptoms and use of diabetic medications and following the American Diabetes Association guidelines as described earlier (21, 22). Nondiabetic controls (N = 1,275) were selected based on a fasting glycemia < 100.8 mg/dl (5.6 mmol/l) or a 2-h glucose < 141.0 mg/dl (7.8 mmol/l). All blood samples were obtained at the baseline visit. Subjects with type 1 diabetes, those with a family member with type 1 diabetes, or rare forms of T2D subtypes (maturity-onset diabetes of the young [MODY]) or secondary diabetes (from, eg, hemochromatosis or pancreatitis) were excluded from the study based on clinical reports, as previously described (20, 22, 24). All participants in this study were recruited following the written informed consent procedures approved by the institutional review boards. All AIDHS/SDS protocols and consent documents were reviewed and approved by the University of Oklahoma Health Sciences Center (OUHSC)’s Institutional Review Board as well as the Human Subject Protection (Ethics) committees at the participating hospitals and institutes in India as described previously (25, 26, 27). The human studies reported in this manuscript abide by the Declaration of Helsinki principles.

BMI was calculated as [weight (kg)/height (m^2^)]. A tape measure of the waist and hip circumferences at the abdomen and the hip, respectively, was recorded. The World Health Organization’s (WHO) new guidelines for the BMI thresholds for Asians were followed (WHO Expert Panel, 2004) (28). Blood pressure (BP) was measured twice after a 5-min seated rest period with the participant’s feet flat on the floor. Serum lipids [total cholesterol, TG, high density lipoprotein–cholesterol (HDL-C), and low density lipoprotein–cholesterol (LDL-C)] were measured using standard enzymatic methods (Roche, Basel, Switzerland) as described previously (22, 29, 30, 31, 32).

### Metabolomics/lipidomics profiling, quality control, and analysis

Aliquots of 50 μl serum/plasma from 3000 AIDHS/SDS individuals were shipped on dry ice to the UC Davis West Coast Metabolomics Center to measure lipidomics profiles using LC-MS/MS in untargeted mode. The serum and plasma aliquots were extracted with a degassed ternary mixture of methanol, methyl tert-butyl ether (MTBE), and water, as detailed before (33). The data produced by LC-MS/MS is a relative qualification done by using internal standards as surrogate markers for quantification. The LC-MS analyses were conducted using an Agilent 1,290 Infinity LC system (Agilent Technologies, Santa Clara, CA) equipped with a Waters Acquity CSH C18 2.1 × 100 mm, 1.7 μm particle size column, maintained at 65°C at a flow-rate of 0.6 ml/min. The mobile phases consisted of (A) 60:40 (v/v) acetonitrile: water with 10 mM ammonium formate/0.1% formic acid and (B) 90:10 (v/v) isopropanol: acetonitrile with 10 mM ammonium formate/0.1% formic acid. Chromatography gradient was: 0 min 15% (B); 0–2 min 30% (B); 2–2.5 min 48% (B); 2.5–11 min 82% (B); 11–11.5 min 99% (B); 11.5–12 min 99% (B); 12–12.1 min 15% (B); and 12.1–15 min 15% (B). Mass spectrometric detection of lipids was performed on Agilent 6530 and 6546 quadrupole time-of-flight mass spectrometers (33) in data-dependent MS/MS mode using both electrospray ionization (ESI) positive and negative ion modes. Raw data were processed using MS-DIAL (v. 4.90) software (34) from 280-1,500 Da from 0.3 min-12.6 min with a centroiding tolerance of 10 mDa and a minimum peak height amplitude of 500 with a mass slice width of 50 mDa, an accurate mass tolerance 10 mDa and 6s alignment retention time tolerance as described (34).

We used three types of internal standards for the analysis, including 43 BioRec (commercial), 43 blank, and 43 pooled samples (derived from representative samples of the AIDHS/SDS population) to assess the technical variance and robustness of the analytical method as described previously (35). Lipids were identified by MS/MS matching to LipidBlast within MS-DIAL at MS1 difference <10 mDa and MS/MS similarity >700 with manual inspection of all similarity matches. All peaks had signal/noise ratios >3:1 for annotated compounds and >10:1 for unknowns using blank samples as negative controls. All data peaks were processed using MS-DIAL 4, and the concentrations (pmol per μl serum or plasma) were calculated by using internal standards, where the tag of level 2 was assigned if the lipid was quantified by an internal standard of the same lipid polar head class, and the tag of level 3 was assigned if the lipid was quantified by an internal standard of a similar lipid class or representative standard compound based on the Lipidomics Standard Initiative (LSI) guidelines (https://lipidomics-standards-initiative.org/) as described previously (34). The identification of unknown MS/MS spectra was elucidated by using internal standards, mining literature, or predicting the putative structure from fragment ion evidence.

### Batch correction

The samples were sent in two batches (N = 1,500/batch) to measure the lipidomics profile. Each batch was sent separately at a different time, with a gap between processing approximately a year. Quality control (QC) was assured by randomizing injection sequences and injecting 10 QC pool samples before the actual sequence of samples, injecting QC pool samples at the beginning and the end of each batch sequence and between each 10 actual samples, injecting QC method blanks after each set of 10 actual samples, checking the peak shape and the intensity of spiked internal standards and the internal standard added before injection. The QC pool samples were used to correct for batch differences, longitudinal drift, and instrument variation, causing technical data variance by random forest machine learning in the SERRF algorithm (36). SERRF defines systematical errors as errors not only associated with batch effects and injection order but also considers patterns of related compounds. The cross-validated relative standard deviation (coefficient of variation, % CV) is used to evaluate the data performance. Per batch, reproducibility was achieved at 7.3% CV as median overall acquired lipids in positive ESI mode using the sample pool QCs. For negative ESI mode, reproducibility was measured at a median 8.6% CV across all lipids for the sample pool QCs.

### Clinical risk score

The CRS for T2D was calculated following the modified version of the Joint British Society risk score as described previously (37, 38). The modified Joint British Society risk factors include age (years) (<30 = 0, ≥30–50 = 1, ≥51–70 = 2, >70 = 3); gender (female = 1, male = 2); BMI (≤23 = 0, 23–27.5 = 1, >27.5 = 2); smoking habits (yes = 1, no = 0); hypertension (hypertensive = 1 [systolic BP (SYSBP) ≥ 110 mmHg and diastolic BP ≥ 90 mmHg], nonhypertensive = 0); an independent assessment of SYSBP (<89 mmHg = 0, 90–130 mmHg = 1. >130 mmHg = 2); family history of diabetes (yes = 1, no = 0); and any other metabolic disorders such as coronary heart disease, arthritis, or kidney disease (yes = 1, no = 0). Note that we use a lower BMI cutoff for defining obesity in South Asians based on the ethnicity-specific guidelines proposed by the WHO (28).

### Statistical analysis

The clinical and demographic variables were summarized using the mean for continuous variables and percentages for categorical variables. Before the statistical analysis, the batch-corrected metabolite profile data were normalized through log transformation, which resulted in some negative values and was scaled using MetaboAnalyst 6.0 (39). The data were divided into three cohorts, namely the discovery cohort consisting of 1,500 individuals sent for LCMS in batch 1, the replication cohort comprising 1,500 individuals for batch 2, and the combined cohort (N = 3,000) containing combined batch-corrected profiles of batches 1 and 2. The orthogonal partial least squares discriminant analysis (OPLS-DA) was used to inspect group disparity. Multivariate linear regression analyses were performed to assess the impact of individual metabolite markers on T2D after adjusting for covariates such as age, gender, BMI, and medications. To understand the effect of gender-based differences in individual lipids, linear regression was done adjusting for age, BMI, and T2D. The effect of CRS on lipids was analyzed through linear regression, adjusting for age. False discovery rate *P*-values were calculated using the Benjamini–Hochberg procedure, accounting for the multiple tests (*P* = 0.05/number of variables) (40). All analyses were performed on normalized metabolite data using SVS version 8.9.1 (Golden Helix, Bozeman, MT; the SPSS software version 29 (IBM, New York City) and Metaboanalyst 6.0 (Canada)).

### Machine learning analysis

Machine learning was used to integrate clinical and metabolomics biomarker data differing among 3,000 individuals by T2D and gender using binary classification method in Python (v3.11.5) using sci-kit-learn (41) and pandas libraries. In binary classification, class labels were determined by T2D cases (N = 1,254 and 471) and non-T2D controls (N = 936 and 339) in serum and plasma, respectively. Similarly, class labels were determined by gender for men (N = 1,223 and 429) and women (N = 967 and 381) in serum and plasma, respectively. The Shapley additive explanations (SHAP) framework from Lundberg (42) was used to identify important features using an XGBoost model with a Python 3 kernel (v3.11.5). Summary plots depict the top influential biomarkers and how they influence the model prediction (43, 44). SHAP uses game theory to assign a value to each feature in a model, and the values are calculated by comparing a model's predictions with and without a particular feature. The SHAP defines the output of the model as a linear sum of the effects of the input features (45). Among the significantly associated metabolites, 847 and 722 metabolites were identified to be associated with T2D and gender, respectively; only those features that were highly dependent on the outcome were selected for this analysis. Selected features were scaled as initial QC to improve the performance and accuracy of machine learning algorithms. Scaling ensures all features contribute equally by converting them to a common scale to eliminate potential biases. The data file was split into 80% training and 20% testing partitions using a defined seed value. Seeds were chosen to ensure that the resulting training and testing accuracy were similar to preventing model overfit. Tenfold cross-validation was used to evaluate the model and detect overfitting. Supervised learning classifiers such as logistic regression (LR), linear discriminant analysis (LDA), K-nearest neighbors classifier (KNN), Gaussian Naïve Bayes (NB), support vector machine (SVM) and a deep learning method, artificial neural network (ANN) were used to classify T2D and gender. The model was evaluated using various classification metrics, such as accuracy, which is defined as the number of correct predictions divided by the total number of predictions. The receiver operator characteristics curve was used as a measure of sensitivity and specificity at different decision thresholds, and the area under the curve (AUC) summarized the overall performance of the model as described previously (46).

## Results

Table 1 shows the clinical characteristics of the study subjects in serum and plasma segregated by T2D status. The average median age of T2D patients was 55.0 and 54.1 years compared to controls 50.0 and 49.0 years in serum and plasma, respectively. As expected, the fasting blood glucose (FBG), waist circumference, TGs, and SYSBP levels were significantly increased in T2D cases compared to controls.

**Table 1:** Clinical characteristics of the AIDHS/SDS individuals with lipidomics data

Trait	Combined (N = 3,000)			Serum (N = 2,186)			Plasma (N = 810)		
	Cases (N = 1725)	Controls (N = 1,275)	*P*-value	Cases (N = 1,254)	Controls (N = 936)	*P*-value	Cases (N = 471)	Controls (N = 339)	*P*-value
Males (%)	922 (53%)	730 (57%)		677 (54%)	541 (58%)		242 (51%)	187 (55%)	
Age (years)	55.0 (16.0)	50.0 (21.2)	1.1 × 10^−24^	55.0 (15.0)	50.0 (21.0)	3.8 × 10^−20^	54.1 (18.0)	49.0 (23.9)	3.6 × 10^−6^
BMI (kg/m^2^)	26.9 (5.8)	26.1 (6.0)	1.0 × 10^−7^	26.8 (5.6)	26.0 (6.2)	1.3 × 10^−6^	26.9 (6.0)	26.3 (5.8)	0.02
Waist (cm)	94.0 (15.2)	91.4 (15.2)	6.1 × 10^−9^	94.0 (15.2)	91.4 (15.1)	5.1 × 10^−6^	94.0 (15.2)	91.2 (15.2)	2.0 × 10^−4^
Waist-to-hip ratio	0.96 (0.1)	0.94 (0.1)	3.5 × 10^−17^	0.96 (0.1)	0.94 (0.1)	1.4 × 10^−10^	0.97 (0.1)	0.94 (0.1)	5.5 × 10^−9^
Systolic BP (mmHg)	143.0 (31.0)	130.0 (25.0)	1.6 × 10^−22^	142.0 (30.0)	130.0 (26.0)	1.5 × 10^−15^	145.0 (34.0)	134.0 (27.0)	1.5 × 10^−9^
Diastolic BP (mmHg)	84.0 (14.0)	80.0 (17.0)	1.8 × 10^−12^	84.0 (13.0)	80.0 (17.0)	1.2 × 10^−11^	85.0 (15.0)	81.5 (16.0)	0.02
Fasting blood glucose (mg/dl)	163.0 (93.0)	97.0 (17.0)	4.2 × 10^−300^	159.5 (88.0)	97.0 (17.0)	9.5 × 10^−218^	169.5 (106.0)	98.0 (17.0)	3.2 × 10^−86^
Age of T2D onset (years)	47.0 (14.0)	-	-	46.1 (14.0)	-	-	47.0 (15.0)	-	-
Triglycerides (mg/dl)	155.0 (106.9)	133.5 (98.0)	1.3 × 10^−6^	157.9 (106.5)	135.0 (97.0)	6.5 × 10^−6^	149.0 (111.0)	131.0 (92.0)	0.06
HDL-C (mg/dl)	37.0 (17.0)	41.0 (17.5)	2.6 × 10^−7^	37.0 (16.0)	41.0 (18.3)	1.6 × 10^−5^	37.0 (19.9)	41.0 (17.0)	0.005
LDL-C (mg/dl)	104.0 (46.5)	109.0 (50.0)	0.0004	103.0 (48.0)	107.5 (49.0)	0.004	106.0 (46.2)	112.5 (52.0)	0.03
Total cholesterol (mg/dl)	175.9 (58.6)	181.3 (62.8)	0.07	174.2 (60.1)	179.5 (64.3)	0.31	177.7 (57.1)	187.7 (58.3)	0.07

Values are displayed in median (IQR).

Men had significantly higher waist-to-hip ratios and higher triglycerides than women, both with and without T2D. Women had significantly higher levels of total cholesterol, HDL-C, and LDL-C than men among both T2D cases and controls (Table 2). Flow chart details the number of specimens used for generating global lipidomics profiles in serum or plasma in the discovery and replication datasets of AIDHS. It also summarizes the number of lipid species identified (Supplemental Fig. S1).

**Table 2:** Clinical characteristics of the AIDHS/SDS individuals with lipidomics data based on gender

Trait	T2D Cases (N = 1725)			Controls (N = 1,275)		
	Men (N = 922)	Women (N = 805)	*P*-value	Men (N = 730)	Women (N = 543)	*P*-value
Age (years)	54.0 (16.0)	55.0 (16.0)	0.15	50.0 (23.7)	49.0 (20.7)	0.20
BMI (kg/m^2^)	26.3 (5.3)	27.5 (6.4)	5.3 × 10^−10^	25.9 (5.2)	26.4 (6.8)	0.02
Waist (cm)	94.0 (15.2)	93.0 (12.7)	0.005	91.4 (15.2)	88.9 (15.2)	1.4 × 10^−10^
Waist-to-hip ratio	0.98 (0.1)	0.94 (0.1)	1.6 × 10^−43^	0.96 (0.1)	0.91 (0.1)	1.6 × 10^−32^
Systolic BP (mmHg)	142.0 (29.0)	144.0 (32.0)	0.36	130.0 (25.0)	128.5 (27.0)	0.13
Diastolic BP (mmHg)	85.0 (13.0)	84.0 (15.0)	0.15	81.0 (16.0)	79.5 (15.0)	6.7 × 10^−4^
Fasting blood glucose (mg/dl)	163.0 (89.0)	162.0 (101.0)	0.77	98.0 (18.0)	97.0 (16.0)	0.69
Age of T2D onset (years)	45.0 (14.0)	48.0 (13.0)	0.006	-	-	-
Triglycerides (mg/dl)	158.0 (116.9)	153.0 (102.0)	0.31	138.5 (106.0)	127.0 (85.3)	0.003
HDL-C (mg/dl)	35.0 (16.0)	39.0 (17.5)	2.5 × 10^−8^	38.0 (16.0)	44.0 (17.5)	1.7 × 10^−12^
LDL-C (mg/dl)	100.0 (46.0)	108.0 (51.3)	5.7 × 10^−6^	105.0 (49.5)	112.9 (49.6)	0.001
Total cholesterol (mg/dl)	172.4 (53.8)	181.3 (60.4)	2.0 × 10^−6^	172.4 (64.5)	188.7 (61.5)	0.001

Values are displayed in median (IQR).

### Altered patterns of circulating lipids in T2D

**Fig. 1:**
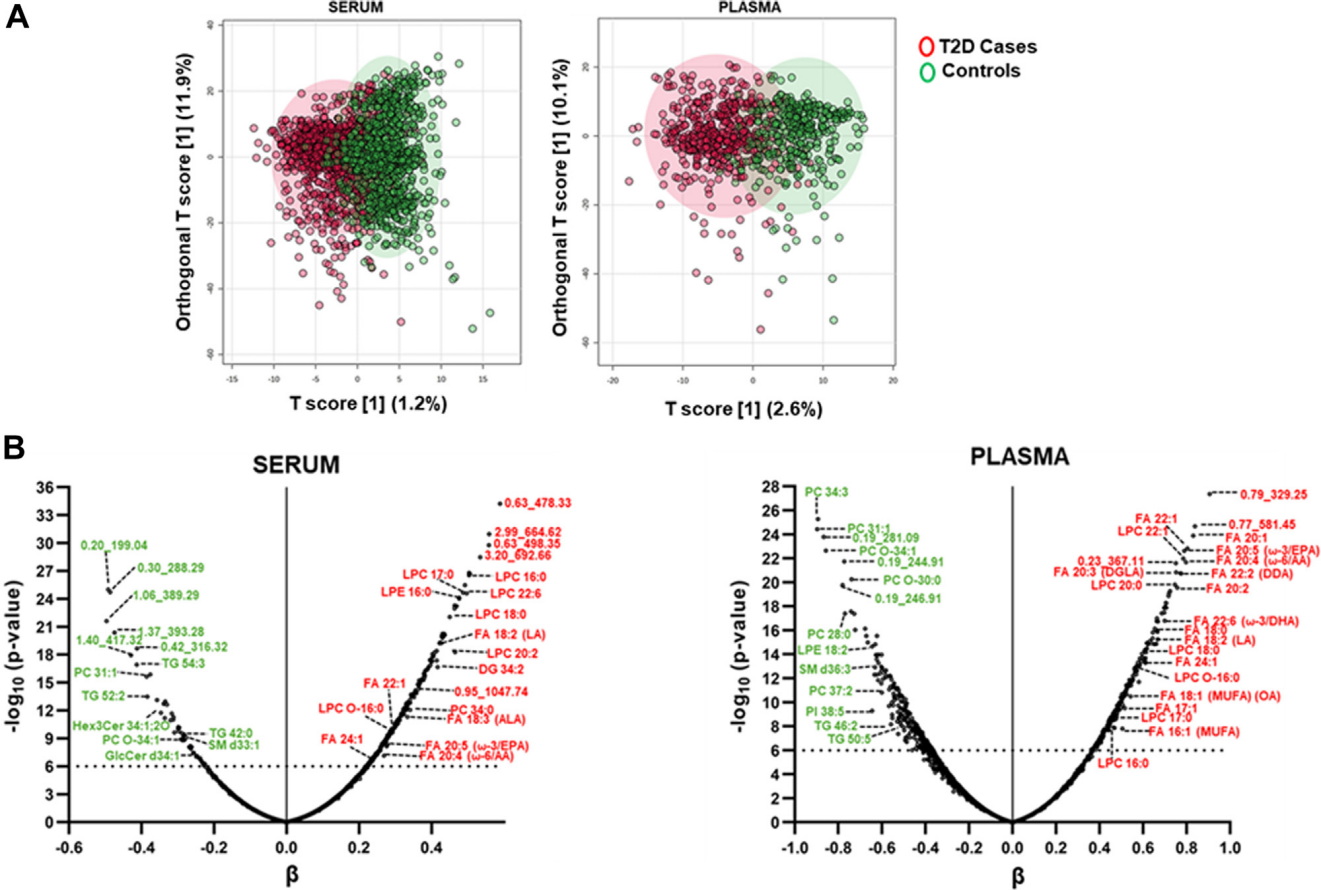
Distribution of circulating lipids by T2D in serum and plasma. A: The orthogonal partial least-squares discrimination analysis (OPLS-DA) with the first principal component and first orthogonal component showed clustering in T2D cases (red) versus controls (green) based on metabolite profiles. Explained variance is shown in brackets for both the Y and X axes. B: Volcano plots represent the significant association of metabolites in T2D cases versus controls in serum and plasma. Red fonts show increased metabolites in T2D with positive regression coefficients and green fonts show decreased metabolites in T2D with negative regression coefficients. T2D, type 2 diabetes.

The OPLS-DA plot with the first principal component and first orthogonal component identified two distinct populations of metabolites, suggesting different lipidomics profiles between T2D cases versus controls (Fig. 1A). Similar lipid regulation patterns were observed in all three sets combined—discovery and replication (Supplemental Figs. S2 and S3). Lipids such as acylcarnitines (CAR) and essential free FAs were significantly upregulated in T2D individuals. Similarly, glycerophospholipids such as lysophosphatidylcholines (LPCs), lysophosphatidylethanolamines, and phosphatidylethanolamines (PEs) were increased, while most metabolite species of PCs were significantly decreased in cases except for PC 34:0 that was significantly upregulated. Sphingolipids such as Cer were significantly increased, whereas glucosylceramide (GlcCer) and most sphingomyelin (SM) were reduced in T2D cases. Diacylglycerols (DGs) and FA-containing TGs showed a 6 to 8-fold increase in people with T2D (Supplementary Fig. S2). DG 34.2 and TG 42.0 remained consistently increased and decreased, respectively, in the sera of T2D patients.

Most metabolites in each lipid class showed a similar regulation pattern in T2D in plasma as observed in serum. Free FAs were significantly upregulated. LPCs and lysophosphatidylethanolamines are significantly increased while PCs are decreased except for PC 34:0. SMs were significantly reduced in T2D cases while saturated Cers (Cer 40:0;2O|Cer 18:0;2O/22:0 and Cer d42:2 Isomer B) were increased. DGs and FA-containing TGs (TG 56:2|TG 16:0_18:1_22:1) are increased, while many TGs with long carbon chains were decreased in T2D individuals in plasma (Supplemental Fig. S3). However, unlike serum, CARs did not differ among T2D and controls, in plasma.

**Fig. 2:**
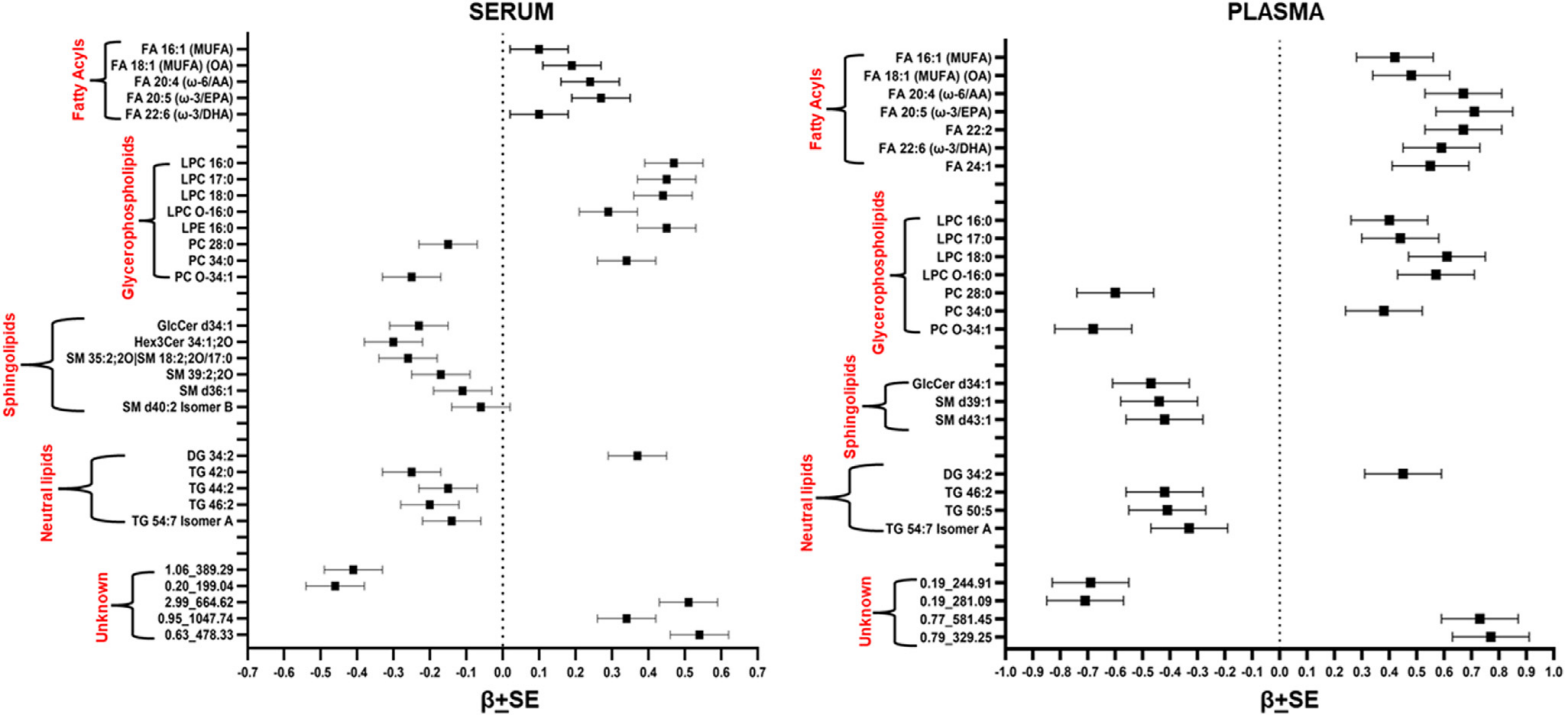
Differences of circulating lipids by T2D in serum and plasma. Forest plots show effect sizes (beta and standard errors) of represented metabolites from major lipid classes significantly associated with T2D in serum and plasma. *P*-values for these associations are mentioned in the supplementary tables.

Multivariate linear regression analysis after controlling for age, gender, BMI or waist, and medication showed MUFAs such as FA 18:1 oleic acid and others. FAs to be significantly increased in serum. Similarly, in plasma FA 16:1 (palmitoleic acid) and FA 18:1 were increased in T2D cases. FA 20:5 (ω-3/eicosapentaenoic acid or EPA) was significantly upregulated in the serum and plasma of diabetic cases. FA 22:6 (ω-3/docosahexaenoic acid or DHA) was also increased in both the serum and plasma of T2D cases (Fig. 2 and Supplemental Table S1). Most of the LPCs are significantly upregulated with LPC 16:0 increased in T2D cases in the serum and plasma. LPC O-16:0 was also significantly upregulated in serum and plasma. PCs were significantly reduced in individuals with T2D except PC 34:0 in both serum and plasma. Sphingolipids such as SMs and GlcCer are reduced in diabetic cases in serum and plasma. Neutral lipids such as DG 34:2 in T2D patients are upregulated in serum and plasma. TGs are significantly reduced in both the serum and plasma of diabetic individuals (Figs. 1B and 2).

### Gender-based differences in circulating lipids in serum and plasma

**Fig. 3:**
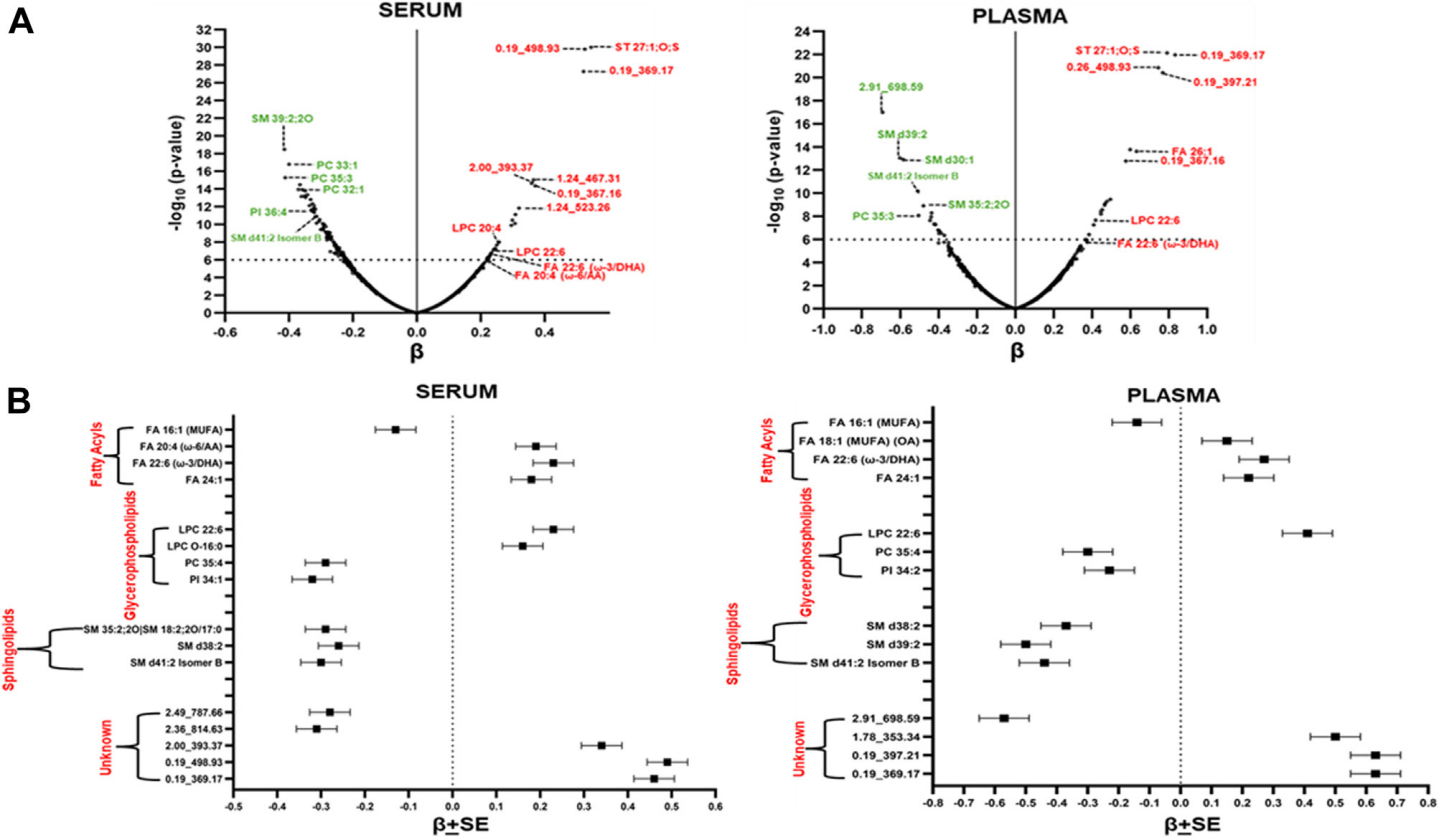
Differences of circulating lipids by gender in serum and plasma. A: Volcano plots represent the significant association of metabolites in serum and plasma in men versus women. B: Forest plots show effect sizes (beta) and standard errors indicating gender-based differences in serum and plasma. *P*-values are mentioned in the supplementary tables.

The OPLS-DA plot with the first principal component and first orthogonal component identified two distinct populations of metabolites, suggesting different lipidomics profiles between men versus women (Supplemental Fig. S4). Free FAs such as FA 24:2 showed a 6-fold increase in serum and a 4-fold increase in plasma in men with *P* = 1.05 × 10^−8^ and 5.33 × 10^−5^ (Supplemental Fig. S5). FA 16:1 palmitoleic acid (ω-7/MUFA) was significantly decreased in both the serum and plasma of men (Fig. 3B). FA 22:6 (ω-3/DHA) was increased in men in both serum and plasma. Similarly, LPC 22:6 showed a 6-fold increase (Supplemental Fig. S5) in the serum and plasma of men when compared to that of women. Most PCs were significantly lowered in men (Fig. 3A, B). SM d38:2 was significantly reduced 5-fold in men with *P* = 5.01 × 10^−8^ and 1.19 × 10^−7^ in serum and plasma. No significant gender-based difference was observed for neutral lipids.

### Inverse relationships of circulating lipids in T2D and obesity and gender differences

**Fig. 4:**
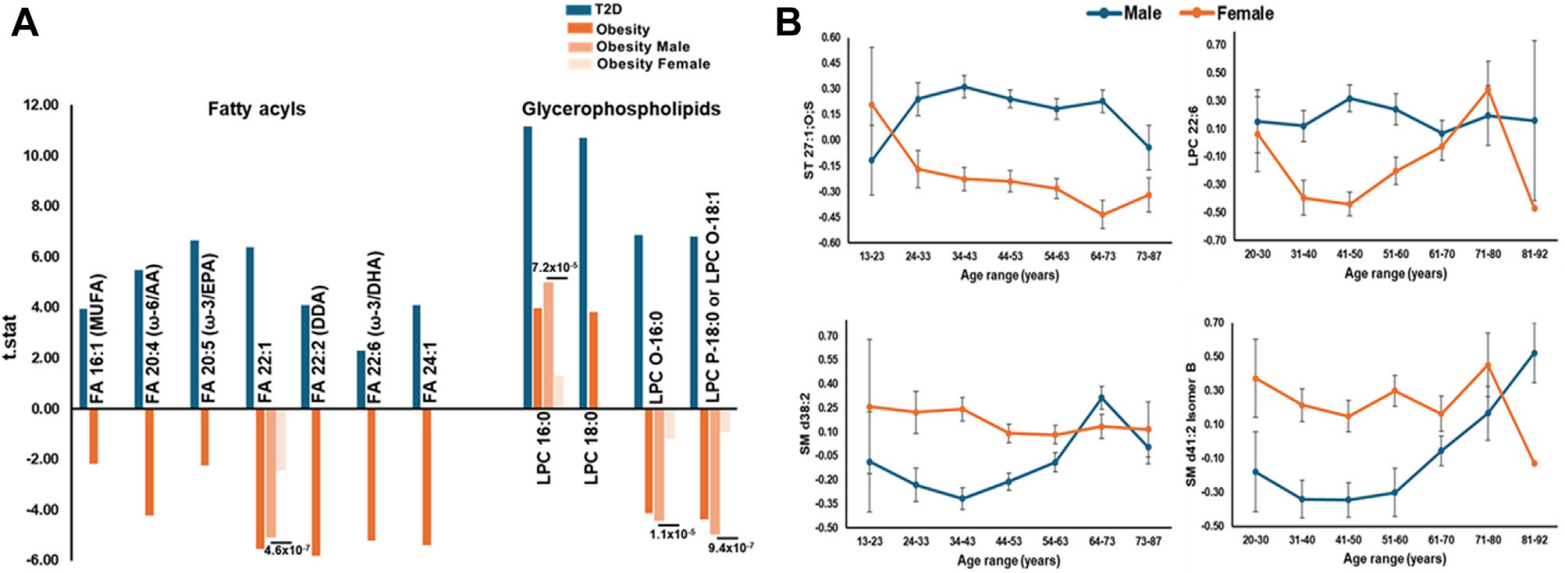
Obesity paradox reveals gender differences due to altered patterns of FAs and LPCs. A: Bar graphs representing the significant differential regulation of different serum lipid classes in T2D cases versus controls and obese versus nonobese individuals. The graph also represents a significant difference between males and females in obese versus nonobese individuals in a few metabolites. B: Line plots depicting the mean expression of metabolites at different age ranges in males and females (values are displayed in mean + SE). FA 16:1 (palmitoleic acid); FA 20:4 (arachidonic acid); FA 20:5 (eicosapentaenoic acid); FA 22:1 (erucic acid); FA 22:2 (docosadienoic acid); FA 22:6 (docosahexaenoic acid); FA 24:1 (nervonic acid); MUFA: monounsaturated fatty acids.

Several free FAs revealed an inverse relationship between T2D and obesity. Essential FAs such as FA 20:4 (ω-6/AA), FA 20:5 (ω-3/EPA), and FA 22:6 (ω-3/DHA) showed a 2 to 7-fold increase in T2D patients, while they were 2 to 5-fold reduced in obese people. FA 16:1 (MUFA), FA 22:2 (docosadienoic acid or DDA), and FA 24:1 (nervonic acid) were upregulated in T2D cases from 4-to-6 fold and decreased 2–6 fold in obese individuals. The gender-based difference was evident in FA 22:1 (erucic acid), with a 5-fold decrease in obese men (*P* = 4.6 × 10^−7^). LPC 16:0 also showed significant gender differences, with obese men showing a 5-fold increase (*P* = 7.2 × 10^−5^) compared to women. LPC O-16:0 and LPC P-18:0 or LPC O-18:1 showed a 7-fold increase in T2D patients but were reduced around 5-fold in obese individuals. Similarly, LPC O-16:0 and LPC P-18:0 or LPC O-18:1 were significantly reduced in obese men by 6-fold (*P* = 1.1 × 10^−5^ and *P* = 9.4 × 10^−7^), respectively (Fig. 4A).

In age-associated analysis, some lipid species including cholesterol sulfate (ST 27:1;O;S), LPC 22:6, and SMs (SM d41:2 Isomer B and SM d38:2) showed gender-specific differences (Fig. 4B). For instance, ST 27:1;O;S remained significantly elevated in men in all age groups compared to women. However, LPC 22:6 was increased in men with T2D but it rapidly increased in women after menopause. Similarly, SM d38:2 and SM d41:2 Isomer B increased sharply in men after their 40s or 50s.

Total body fat percentage was significantly higher in women than men, irrespective of T2D (*P* = 6.2 × 10^−11^ T2D and *P* = 8.9 × 10^−6^ controls) (Supplemental Fig. S6A). In gender-stratified data, men showed an insignificant but consistently reduced (EPA/AA) ratio compared to women irrespective of T2D (Supplemental Fig. S6B).

### Association of circulating lipids with CRS

The CRS was robustly associated with T2D among men and women (Supplemental Table S2). CRS showed a similar pattern of association as T2D in both serum and plasma. In serum, lipids such as CAR 12:0; CAR 14:2; FA 18:2 (linoleic acid or LA), and FA 20:5 (ω-3/EPA) were upregulated. Glycerophospholipids such as LPCs, LPC 16:0 and LPC 22:6 were also increased in people with high CRS. PCs like PC 31:1, oxidized PC O-32:0 showed a significant reduction in high CRS individuals. Sphingolipids such as GlcCer d34:1 and SM d36:1 decreased in the high CRS group (Supplemental Fig. S7). Plasma lipids including CAR 12:0; CAR 14:2; FA 18:2 (LA), and FA 20:5 (ω-3/EPA) were upregulated. Glycerophospholipids such as LPCs, LPC 16:0 and LPC 22:6 were also increased in people with high CRS. PCs like PC 31:1, oxidized PC O-32:0 showed a significant reduction in high CRS individuals. Sphingolipids such as GlcCer d34:1 and SM d33:1 decreased in the high CRS group (Supplemental Fig. S7).

### Machine learning analysis for identifying metabolite features associated with T2D

**Fig. 5:**
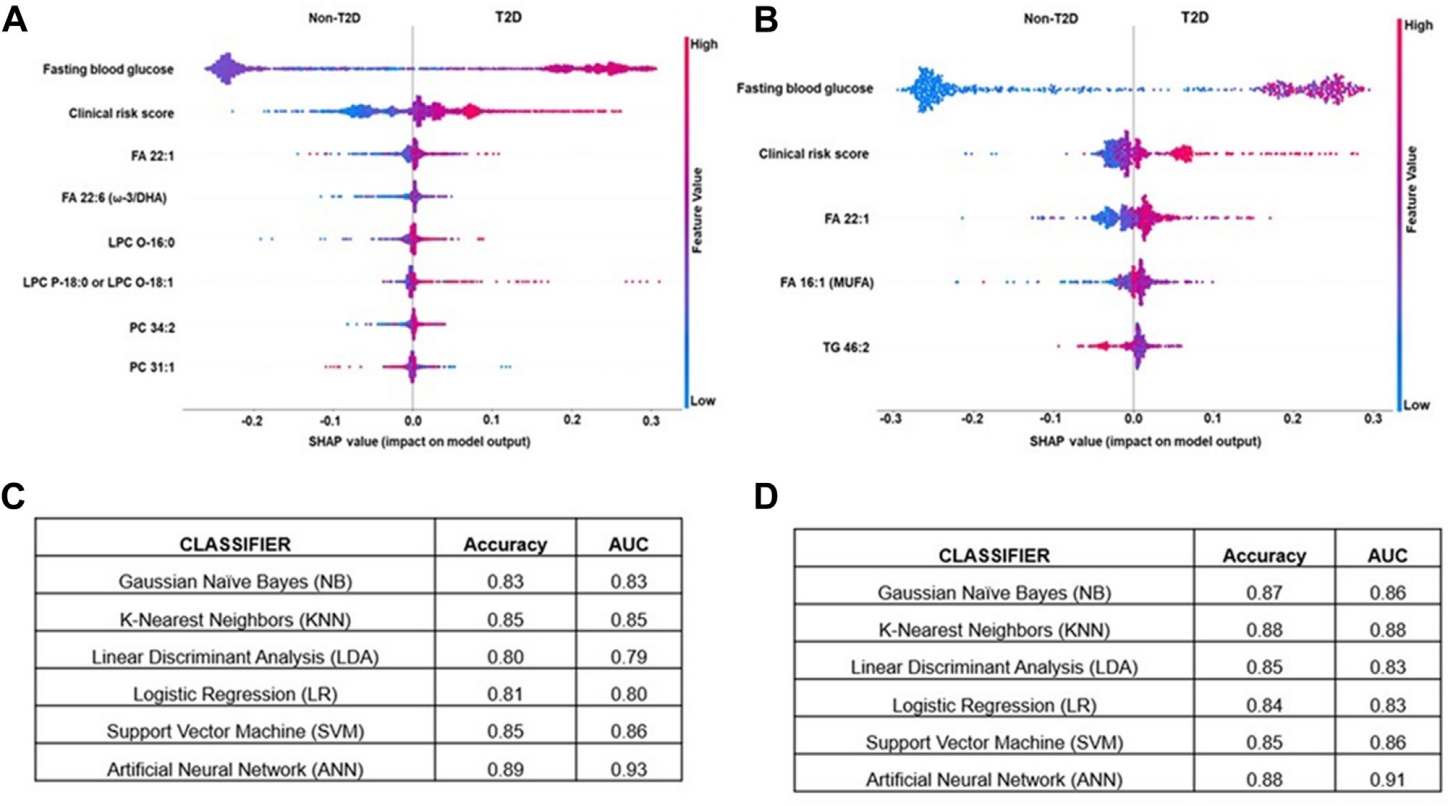
Identification of predictive features of T2D using machine learning analysis. A: The most important predictive parameters using binary classification with T2D, the absolute value of a feature being high (red) or low (blue), depicting T2D or non-T2D in serum. B: The most important predictive parameters using binary classification with T2D, the absolute value of a feature is high (red) or low (blue), depicting T2D or non-T2D in plasma. C: Machine learning analysis for clinical and metabolite features using binary classification and artificial neural networks was the most informative model, showing high sensitivity and specificity in AUC in serum. D: Machine learning analysis for clinical and metabolite features using binary classification and artificial neural networks was the most informative model, showing high sensitivity and specificity in AUC in plasma. T2D, type 2 diabetes.

The clinical and metabolic markers that showed the strongest association were utilized in the final analysis for identifying biomarkers differentially regulated in T2D cases using machine learning analysis. Feature selection using SHAP identified 8 and 5 interactive features, respectively, for serum and plasma, with a higher impact on the model. We identified 6 metabolites (FA 22:1; FA 22:6 (ω-3/DHA); LPC O-16:0; LPC P-18:0 or LPC O-18:1; PC 34:2; PC 31:1) and clinical parameters such as FBG, CRS as the most informative features for serum, as detailed in Figure 5A. Similarly, the features in plasma included clinical parameters such as FBG, CRS, and three metabolites (FA 22:1; FA 16:1 (MUFA); TG 46:2) (Fig. 5B). The combined list of features was used in tenfold cross-validation. For serum, ANN was the most powerful classifier, with an accuracy of 0.89 and an AUC of 0.93. It was followed by SVM with an accuracy of 0.85 and an AUC of 0.86, KNN with an accuracy of 0.85 and an AUC of 0.85, NB with an accuracy of 0.83 and an AUC of 0.83, LR with an accuracy of 0.81 and an AUC of 0.80, and LDA with an accuracy of 0.80 and an AUC of 0.79 (Fig. 5C). For plasma, ANN was the most powerful classifier, with an accuracy of 0.88 and an AUC of 0.91. It was followed by KNN with an accuracy of 0.88 and an AUC of 0.88, NB with an accuracy of 0.87 and an AUC of 0.86, SVM with an accuracy of 0.85 and an AUC of 0.86, LDA with an accuracy of 0.85 and an AUC of 0.83, and LR with an accuracy of 0.84 and an AUC of 0.83 (Fig. 5D).

### Machine learning analysis for identifying metabolite features associated with gender

**Fig. 6:**
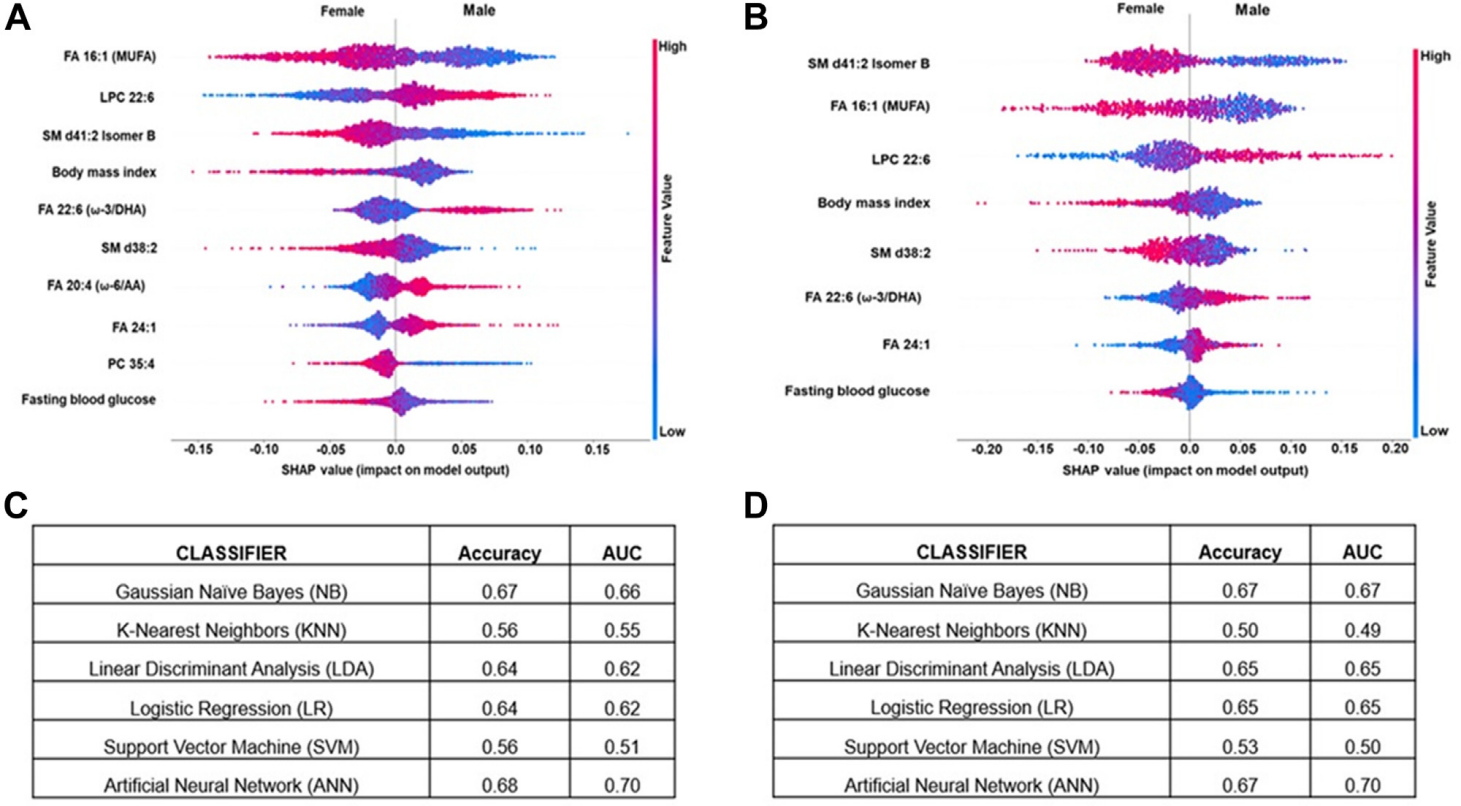
Identification of predictive features associated with gender in machine learning analysis. A: The most important predictive parameters using binary classification with gender, the absolute value of a feature being high (red) or low (blue) depicting male or female in serum. B: The most important predictive parameters using binary classification with gender, the absolute value of a feature is high (red) or low (blue), depicting male or female in plasma. C: Machine learning analysis for clinical and metabolite features using binary classification and artificial neural networks was the most informative model, showing high sensitivity and specificity in AUC in serum. D: Machine learning analysis for clinical and metabolite features using binary classification and artificial neural networks was the most informative model, showing high sensitivity and specificity in AUC in plasma.

The clinical and lipid markers that showed the strongest gender-based association were utilized in the machine learning models for further identifying the most informative features underlying gender differences. Using SHAP analysis, we identified 10 and 8 interactive features that had the highest impact on the models in serum and plasma. The features in serum included clinical parameters such as FBG, BMI, and eight metabolites (FA 16:1(MUFA); LPC 22:6; SM d41:2 Isomer B; FA 22:6 (ω-3/DHA); SM d38:2; FA 20:4 (ω-6/AA); FA 24:1; PC 35:4), as detailed in Figure 6A. The features in plasma included clinical parameters such as FBG, BMI, and six metabolites (SM d41:2 Isomer B; FA 16:1(MUFA); LPC 22:6; SM d38:2; FA 22:6 (ω-3/DHA); FA 24:1), as detailed in Figure 6B. The combined list of features was used in tenfold cross-validation. The ANN was the most powerful classifier, with an AUC of 0.70, in serum and plasma, while other models showed modest AUC and accuracy (Fig. 6C, D).

## Discussion

The role of free FAs in T2D, as well as their dietary intake from various food groups (as FA pools), has been a significant topic of discussion over the past two decades. This is largely due to conflicting reports from numerous cohort and observational studies, particularly about the functional roles of various essential FAs, such as ω-3/n-3 and ω-6/n-6 types. Notably, the most FAs found significant with T2D are the free FAs that travel in plasma/serum as part of the albumin complex and are generally released from adipocytes. While free FAs can also be hydrolyzed from other complex lipids, e.g., PCs, phosphatidylethanolamines, or even TGs, their contribution from these sources is generally minor. In contrast, many other publications report on “total fatty acids,” ie, the lipidome's total FA content, which is mainly driven by the hydrolysis of dietary TGs. These differences may have contributed to the differences behind the conflicting results.

Even though many studies have indicated that ω-3 and ω-6 MUFA and PUFA can protect against T2D, many other studies report the opposite effects. Here, our data did not confirm the beneficial effects of several known FAs for decreasing T2D risk. Rather, we observed a significant increase of FA 20:4 (ω-6/AA), FA 20:5 (ω-3/EPA), and FA 22:6 (ω-3/DHA) in patients with T2D even after adjusting for age, gender, and BMI. We observed the highest OR of FA20:5 (ω-3/EPA) for increasing the risk for T2D in both serum and plasma of all FAs. Somewhat similar results have been reported by other T2D studies (47, 48). Saturated FAs (16:0, 18:0), and unsaturated FAs (16:1, 18:1,18:3, 20:3, and FA22:6 (ω-3/DHA) are also increased in lung cancer patients (49, 50).

Like many other studies, most saturated and unsaturated PCs were negatively associated with T2D, except PC 34:0, and PC 34:2 were increased in T2D patients in this study. However, levels of several saturated LPCs (LPC 16:0, LPC 18:0), many MUFA and PUFA-containing LPCs, including LPC 22:5 (EPA), and ether-containing LPC (LPC-O 16:0) were increased and strongly associated with increased risk of T2D. The LPCs are produced by the turnover of PC by phospholipase A_2_ (PLA_2_). Overexpression of PLA_2_ increases LPC content in oxidized LDL, which leads to endothelial dysfunction and atherosclerotic disease (51). Many long carbon chains containing TG species (TG 42:2, TG 46:2, TG 50:5, and TG 54:7 isomer A) were significantly reduced in T2D. Interestingly, the pattern of alteration of FAs, PC, LPCs, and FAs correlated similarly with CRS as with T2D, which further supports the role of these lipids in T2D etiology.

Many metabolites showed a similar association pattern in serum and plasma. Sex differences were significant in many essential FAs in both serum and plasma, where ω-6 MUFA (FA 18:1) and PUFA, including (FA20:4 AA/ω-6) and FA22:6 DHA/ω-3) were significantly increased in men compared to women. Likewise, LPC containing FA22:6/DHA levels were significantly increased in men.

We further explored whether any of these observed gender differences were due to underlying obesity. Indeed, the blood levels of most ω-3 and ω-6 essential FAs were reduced by 2 to 6-fold in obesity in both genders. Saturated species of LPCs (LPC16:0, LPC18:0), as well as ether or plasmalogen-containing LPCs, were significantly reduced in obesity. However, ether or plasmalogen-containing LPCs (LPC-O-16:0 and LPC P-18:0 or LPC O-18:1) were reduced in obesity only in men compared to women, suggesting moderate obesity might protect men from T2D-related outcomes. LPC levels are generally reduced in obesity in both humans and animals (52). Some studies indicate lower LPC levels in insulin resistance (53, 54).

Gender differences in obesity-related diseases have been partly explained by the distribution of adipose fat accumulation rather than overall body fatness. Visceral fat accumulation is linked to worse CVD outcomes, while subcutaneous fat is associated with a lower risk of such outcomes. In this study, total fat percentage was significantly higher among women than men, irrespective of T2D. However, regardless of fat percentage, lipid metabolism is different in men and women in this study, as supported by earlier studies (55, 56). Therefore, more research is needed to understand the complex effects of obesity on circulating lipids and their role in T2D by gender.

The ω-3 FAs are formed from α-linolenic acid (ALA, 18:3), and ω-6 FAs are derived from linoleic acid (LA, 18:2). LA is converted to arachidonic acid (AA, 20:4 ω-6), and ALA is transformed into EPA (20:5) and DHA (22:6); both ω-3 FAs. Upon their digestion, FAs are subsequently converted to long-chain (>18) or very long-chain FAs (>20) by an enzyme elongase, and a degree of unsaturation is achieved through the enzyme fatty acid desaturase (FADS 1, 2) (Supplemental Fig. S8). A ratio of (EPA, 20:5 ω-3)/(AA, 20:4 ω-6) is considered a marker for the anti-inflammatory role of FAs in CVD based on the 2019 European Society of Cardiology and European Atherosclerosis Society guidelines (57). The reduced ratio is linked with metabolic dysfunction, but the increased ratio is considered to prevent CVD. The unbalanced (EPA/AA) ratio in favor of ω-6 FAs promotes inflammation, thrombosis, insulin resistance, T2D, and myocardial infarction in many studies (58). This is due to the possible increase of AA eicosanoids such as prostaglandin E2 and leukotriene (LT) B4- known markers for inflammation and thrombosis. Thus, the unbalanced (EPA/AA) ratio may explain the higher incidence and mortality due to myocardial infarction and coronary artery disease (CAD) in Asian Indian men (7, 59).

Several species of medium to long carbon chain-containing PCs and SMs (>C14) were substantially reduced in men compared to women. PCs 34:1 and 35:4 were decreased significantly in men, showing agreement with earlier published studies (60). Decreased SMs and PCs have been shown to increase the risk for T2D, myocardial infarction, and ischemic stroke (61, 62, 63, 64). Cers and SMs are metabolically interconnected and highly bioactive signaling compounds. SMs are located in cell membranes and lipid-rich lipoproteins and also play an important role in the insulin signaling pathway linked to T2D and related microvascular and macrovascular complications (64, 65). The metabolism of SM is enzymatically regulated and genetic variation impacting enzyme activity may be partially responsible for the reduced SM metabolism and subsequent increased risk for T2D and CVD (61, 66). Moreover, South Asian men have up to 3-fold higher risk for CAD than European men, and South Asian women have a slightly lower prevalence of CAD compared to South Asian men (67).

A significant upward trajectory was observed for the sterol metabolite ST 27:1;O;S, which is cholesterol substituted by a sulfoxide group at position 3, remained significantly elevated in men at all age groups compared to women, and could become a potential biomarker for explaining the increased risk for CVD in men. Sterol sulfonation is tightly linked to cholesterol homeostasis; however, the dysregulation of the SULT2B1b enzyme, which is crucial for cholesterol sulfuration, can lead to abnormal cholesterol metabolism and contribute to the progression of diseases like certain cancers, Alzheimer's disease by enhancing the development of amyloid beta plaque (68). Further studies are necessary to confirm its role in CVD concerning gender differences LPC 22:6 (with DHA FA) increased in men with T2D, and rapidly increased in women after menopause, may increase their risk for CAD after menopause (67). Our data showed women generally had higher circulatory SMs at all ages, and lower levels were associated with T2D in both genders. Many SMs and SM-derived Cers increase with age and have been reported to accumulate in aged muscles, affecting functional capacity, including heart muscles (69). A higher number of carbon chain-containing SMs sharply increased in men after their 40s or 50s, which may explain the gender-associated early age-related CAD increase in South Asian men (70). Age-related trajectories of sphingolipids in men and women have been found in Dutch and South Asians living in the Netherlands (62). However, gender-based elevation in cholesterol sulfate and LPC 22:6 has not been reported in any study. PLA_2_-mediated conversion of PC into LPC increases AA (FA 20:4), which gets bio-transformed to leukotriene B4 and is linked to erectile dysfunction (ED) (71). Testosterone deficiency and ED correlate strongly in men with T2D. The vast majority of our male T2D patients (age >45) in this cohort self-reported having ED. We earlier reported gender differences in vitamin D in this cohort, where circulating 25(OH)D levels remained significantly reduced in men in comparison to women with T2D and obesity (72). Furthermore, our study of telomere length in this cohort revealed that Asian Indian men had significantly shorter telomere length compared to Asian Indian women, and shorter telomere length was associated with a significantly increased risk of cardiometabolic diseases (22).

One explanation for the gender-specific association in this population could be the lifestyle differences in terms of alcohol intake among men and women. Indian women, in general, do not consume alcohol due to cultural and religious reasons (20). Also, a vast majority of our participants were not on hormonal therapy. The differences in blood lipids, particularly higher levels of HDL lipoprotein cholesterol in women than men, and other physiological modulators may influence these differences (30). Despite these differences in men, the global prevalence rates of CAD-related mortality, particularly among Asian Indians, are not entirely explained by lifestyle differences, indicating a need for deeper biological explanations. These results underscore the importance of studying gender differences as biological differences (hormones) and genetic variation, specifically in X and Y chromosomes, can significantly influence the progression and severity of disease, despite the influence of other common factors.

Machine learning analysis for T2D identified 6 metabolite features (FA 22:1, FA 22:6 (ω-3/DHA), LPC O-16:0, LPC P-18:0 or LPC O-18:1, PC 34:2, and PC 31:1) to be the most important markers in individuals with T2D in addition to CRS and FBG in serum and three top features (FA 22:1, FA 16:1, and TG 46:2) were the top metabolites along with CRS and FBG in plasma showing differential patterns in T2D. Our machine learning analysis further validated the role of FAs (MUFA and PUFA ω-3 and ω-6 essential FAs) and LPC 22:6 with SM d38:2 and SM d41:2 Isomer B, revealing gender differences and their susceptibility to T2D. The modest AUC of 0.70 suggests that future genetic studies will be crucial to pinpointing the underlying mechanism of gender differences.

This study has several strengths and weaknesses. The primary strength of this study is that it is the first large-scale, comprehensive report examining the association between the global lipidome and T2D in a well-characterized population from North India. Second, the metabolomic profiles were rigorously quality-checked for batch differences, longitudinal drift, and instrument variation that can cause technical inconsistencies. These corrections were achieved using supervised machine-learning random forest algorithms, as detailed in the methods section. Third, data analysis was conducted using various distinct analysis platforms to ensure reproducibility through rigorous QC and analysis using discovery and validation sets. Weaknesses include a lack of data from other Asian Indian populations for independent validation, specifically for confirming the positive association of ω-3 and ω-6 MUFA and PUFAs with T2D. Even though similar findings have been reported in some earlier studies, more studies would be needed to clarify the inconclusive and controversial role of these essential FAs in human metabolic diseases.

In conclusion, our study could not confirm the protective effects of essential ω-FAs against T2D. The conflicting findings may stem from the variation induced by dietary sources, the types of FAs consumed, the presence of other dietary components that interact with FA metabolism, and individual metabolic responses. These findings underscore the need for future research to clarify these relationships to determine specific dietary recommendations for individuals at risk for or diagnosed with T2D. Moreover, the gender-based alterations in T2D and obesity observed in our study may suggest the underlying mechanism of metabolic and CVD development, which needs further investigation.

## Data availability

Available on request through collaborations.

## Supplemental data

This article contains supplemental data.

## Conflict of interest

The authors declare that they have no conflicts of interest with the contents of this article.
