# Supplementary material for: Circulating lipidome underpins gender differences in the pathogenesis of type 2 diabetes
Source: J Lipid Res. 2025 Apr 26;66(6):100816. doi: 10.1016/j.jlr.2025.100816 (PMC12155659; doi:10.1016/j.jlr.2025.100816)
Supplement: Supplementary files [file mmc2.docx]

**Supplementary Table 1:** The association of fatty acids with T2D

|  | **SERUM** | | | | **PLASMA** | | | |
| --- | --- | --- | --- | --- | --- | --- | --- | --- |
| **Metabolite** | **Beta^** | **SE** | **P-Value** | **FDR** | **Beta^** | **SE** | **P-Value** | **FDR** |
| FA 16:1 (MUFA) | 0.11 | 0.05 | 2.44E-02 |  | 0.37 | 0.08 | 2.26E-06 | ** |
| FA 18:1 (MUFA) (OA) | 0.21 | 0.05 | 9.49E-06 | ** | 0.45 | 0.08 | 8.72E-09 | ** |
| FA 20:4 (ω-6/AA) | 0.23 | 0.05 | 1.30E-06 | ** | 0.51 | 0.07 | 1.34E-11 | ** |
| FA 20:5 (ω-3/EPA) | 0.23 | 0.05 | 2.39E-06 | ** | 0.56 | 0.07 | 1.60E-13 | ** |
| FA 22:1 | 0.27 | 0.05 | 2.42E-08 | ** | 0.67 | 0.08 | 1.01E-17 | ** |
| FA 22:2 (DDA) | 0.21 | 0.05 | 1.38E-05 | ** | 0.62 | 0.08 | 1.03E-15 | ** |
| FA 22:6 (ω-3/DHA) | 0.13 | 0.05 | 4.95E-03 |  | 0.48 | 0.07 | 2.82E-10 | ** |
| FA 24:1 | 0.23 | 0.05 | 2.27E-06 | ** | 0.52 | 0.07 | 4.50E-11 | ** |

^adjusted for covariates age, gender, BMI, and medications; **FDR p for serum 1.2x10^-4^ (0.05/411 metabolites+covariates),

FDR for plasma 1.1x10^-4^ (0.05/447 metabolites+covariates)

**Supplementary Table 2:** The association of clinical risk scores with T2D

| **Cohort** | **β** | **SE** | **OR** | **LCI (95% CI)** | **UCI (95%CI)** | **P-value** |
| --- | --- | --- | --- | --- | --- | --- |
| Combined | 2.10 | 0.07 | 8.17 | 8.03 | 8.30 | 1.36E-190 |
| Male | 2.02 | 0.09 | 7.54 | 7.36 | 7.71 | 3.54E-107 |
| Female | 2.29 | 0.10 | 9.87 | 9.68 | 10.07 | 6.17E-103 |

OR: odds ratio; LCI: Lower confidence interval; UCI: Upper confidence interval;


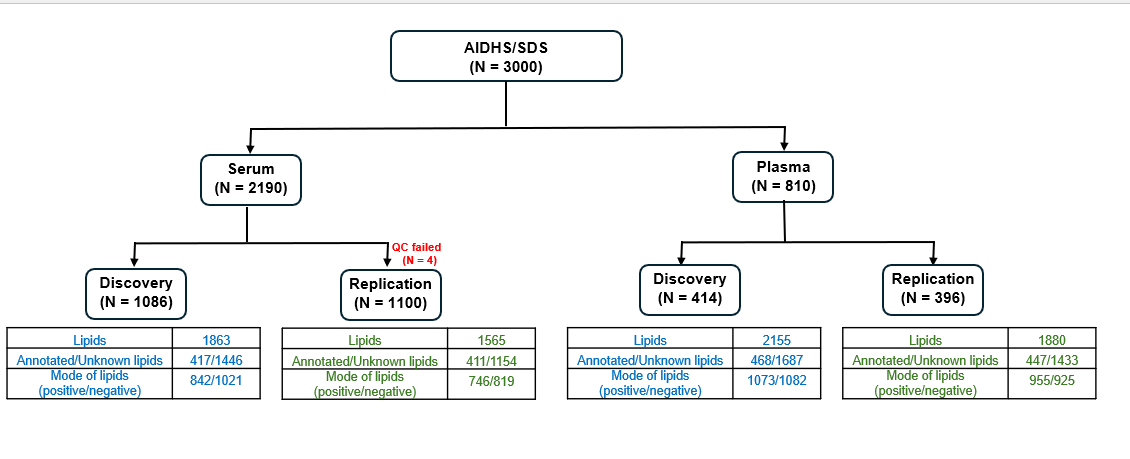
 **Supplementary Figure 1**: Flow chart describes the number of specimens used for generating global lipidomics profiles using serum or plasma in discovery and replication datasets of AIDHS. Lower charts show the total number of metabolites identified, the number of annotated, and unknown metabolites in +ve and -ve modes.


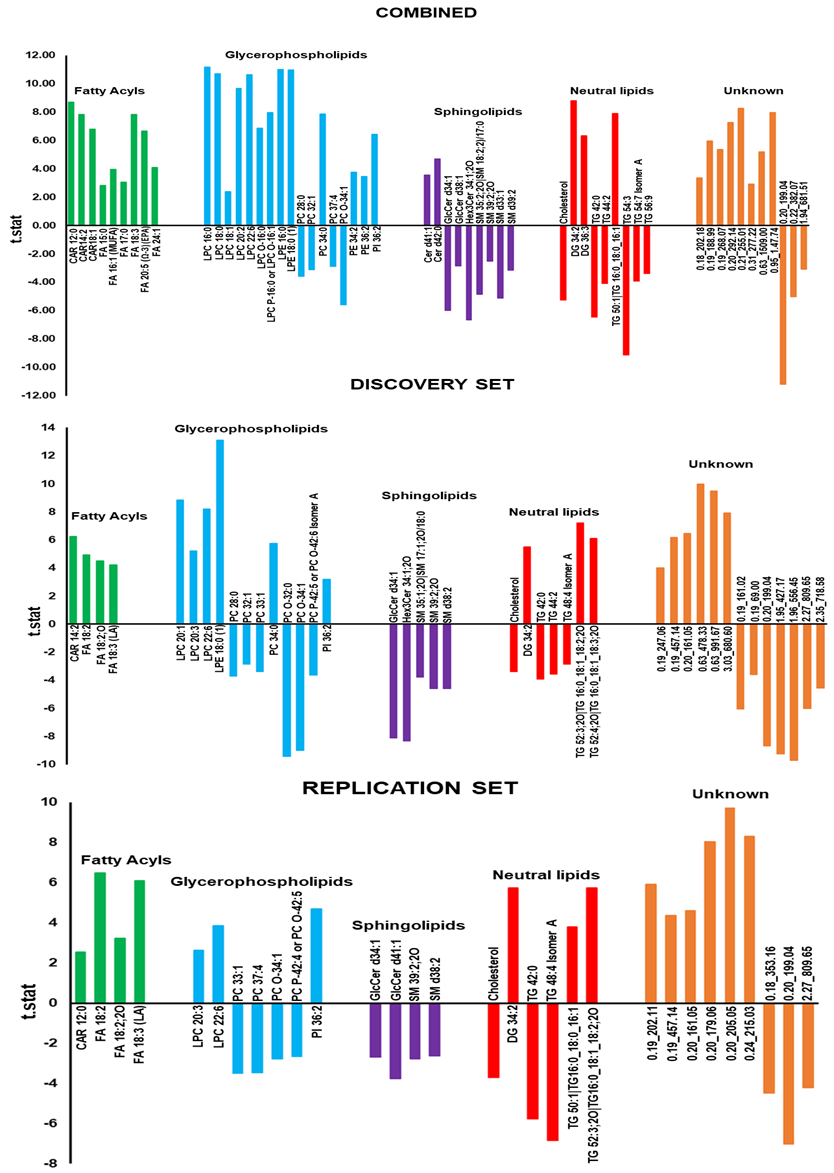


**Supplementary Figure 2**: Bar graphs represent the differential regulation of different serum lipid classes in T2D cases vs. controls in combined, discovery, and replication sets.


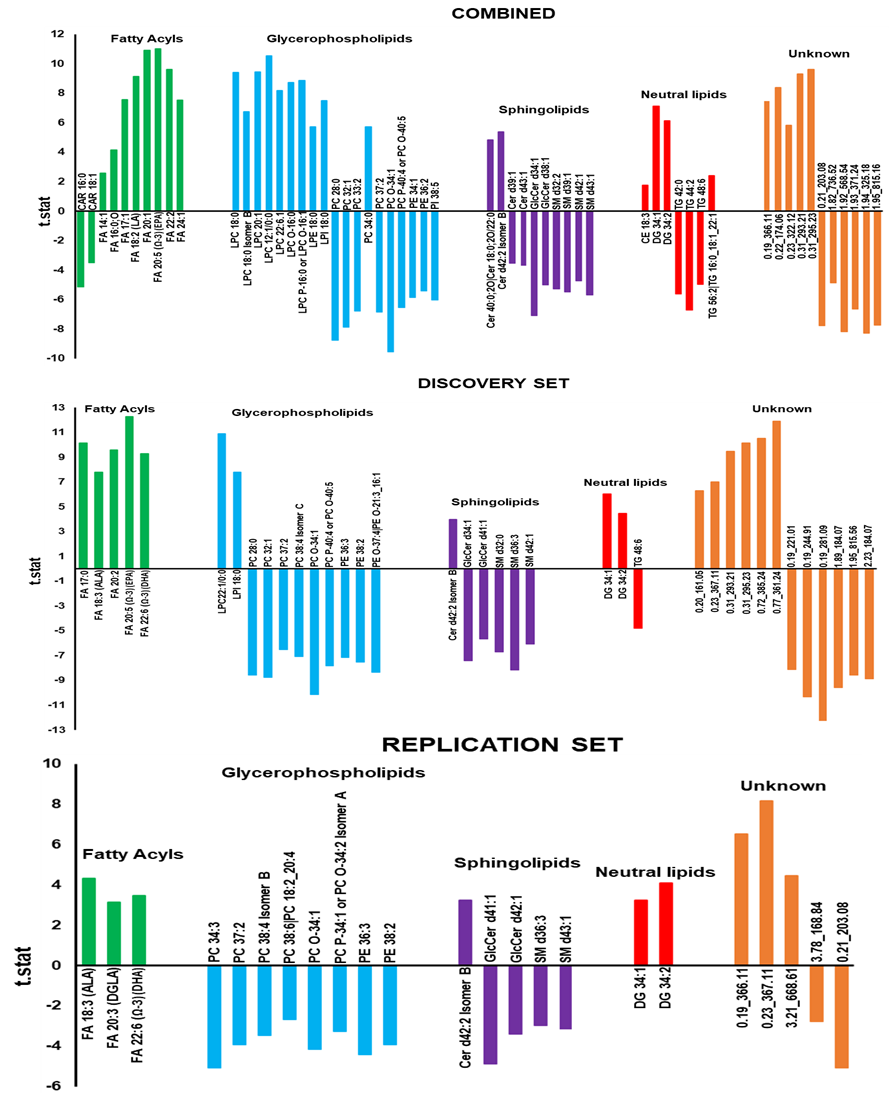


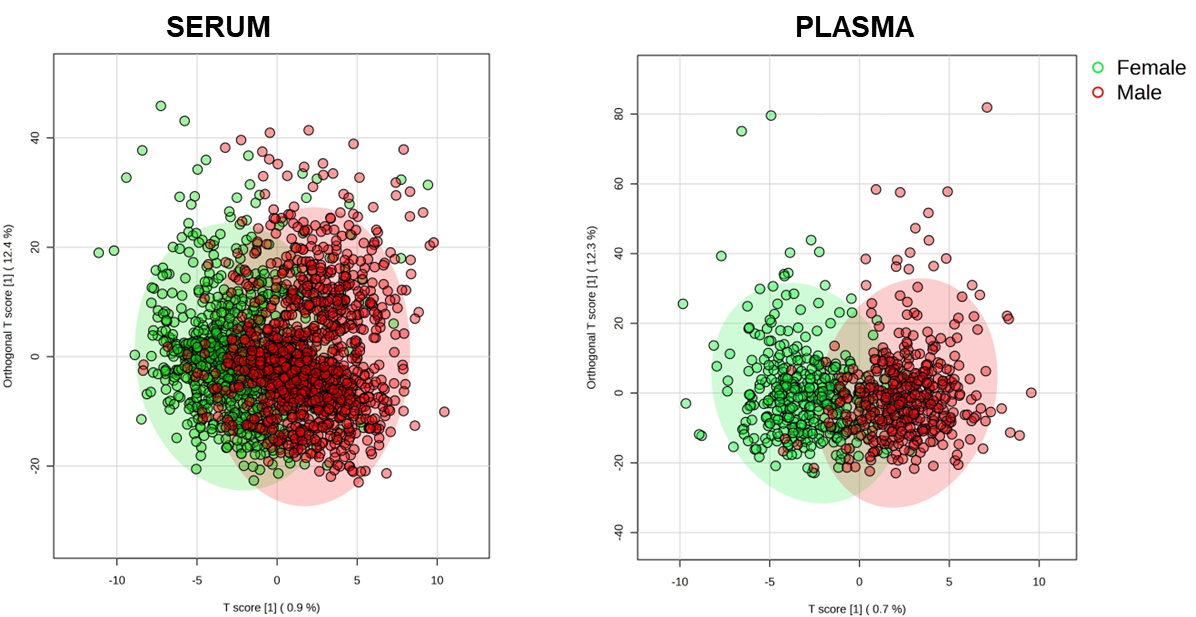


**Supplementary Figure 3**: Bar graphs represent the differential regulation of different plasma lipid classes in T2D cases vs. controls in combined, discovery, and replication sets.

**Supplementary Figure 4**: The Orthogonal Partial Least-Squares discrimination analysis (OPLS-DA) with the first principal component and first orthogonal component showed clustering in Male (red) vs. Female (green) based on metabolite profiles. Explained variance is shown in brackets for both the Y and X axes.


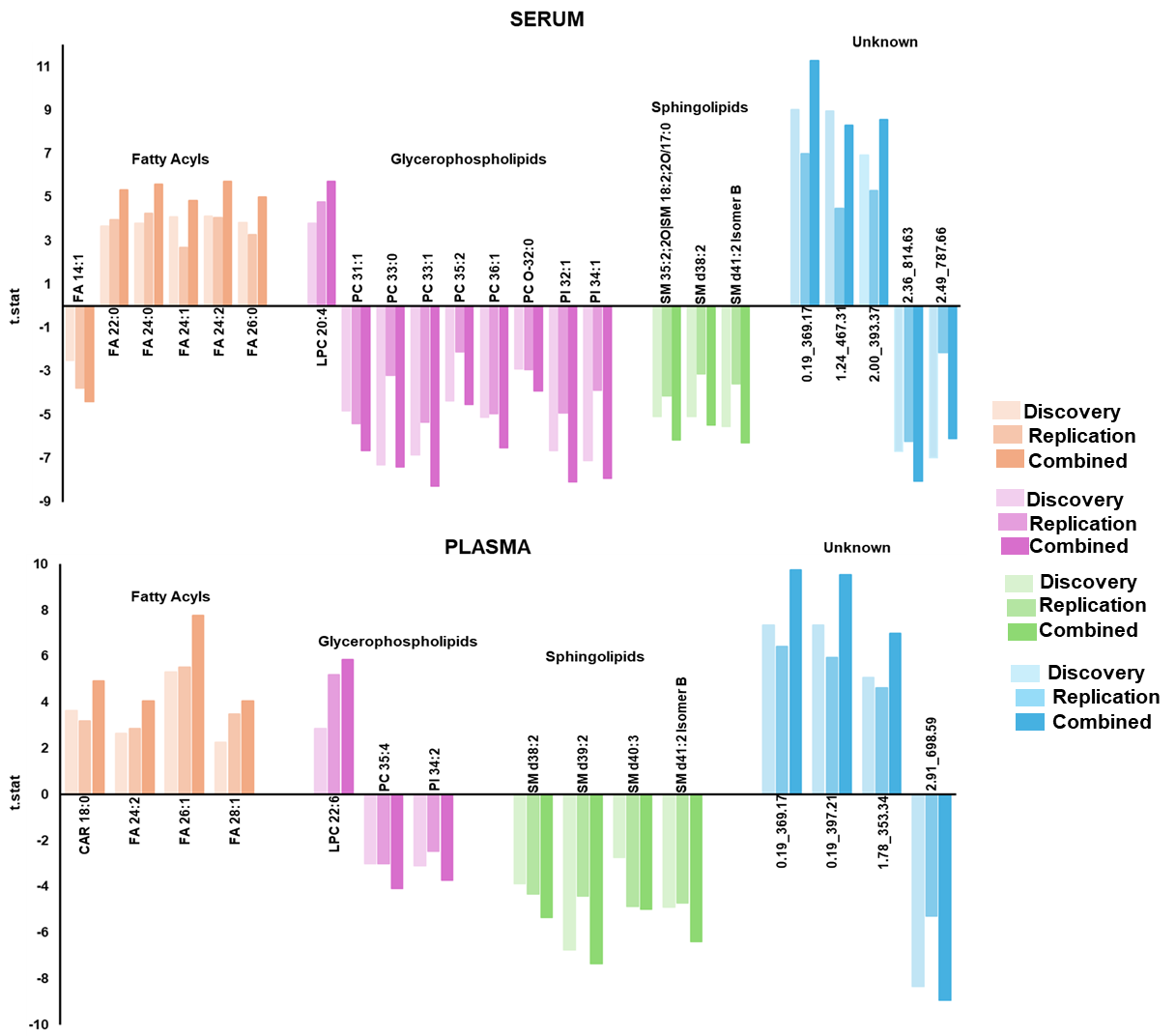


**Supplementary Figure 5**: Bar graphs represent the differential regulation of metabolites in serum and plasma in four lipid classes by gender (men vs. women) in discovery, replication, and combined datasets.

**Supplementary Figure 6**: Bar plot depicting the average (**A**) Fat (%) by gender and (**B**) Gender difference in the ratio of FA 20:5 (ω-3/EPA)/FA 20:4 (ω-6/AA) metabolites in association with T2D in combined, men and women.

Values are displayed in mean+SE


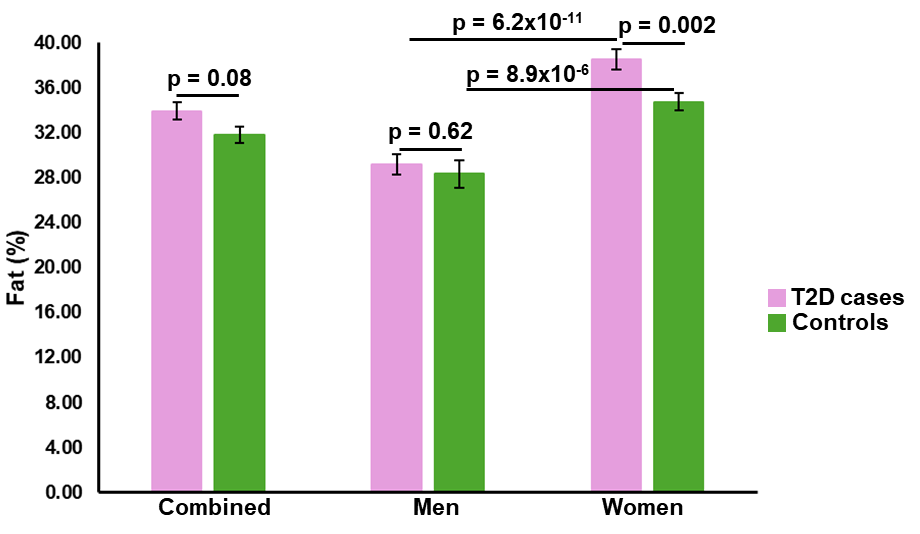

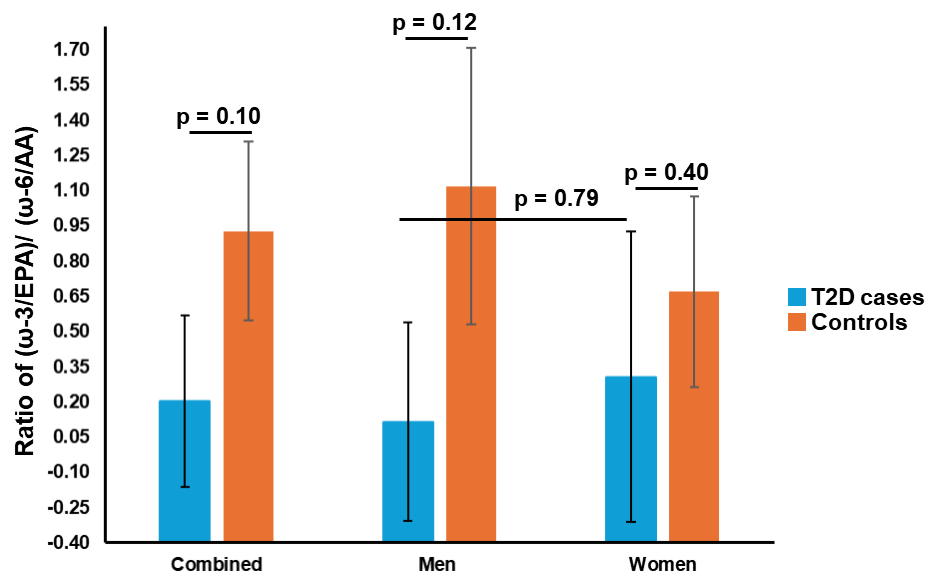


**A**

**B**


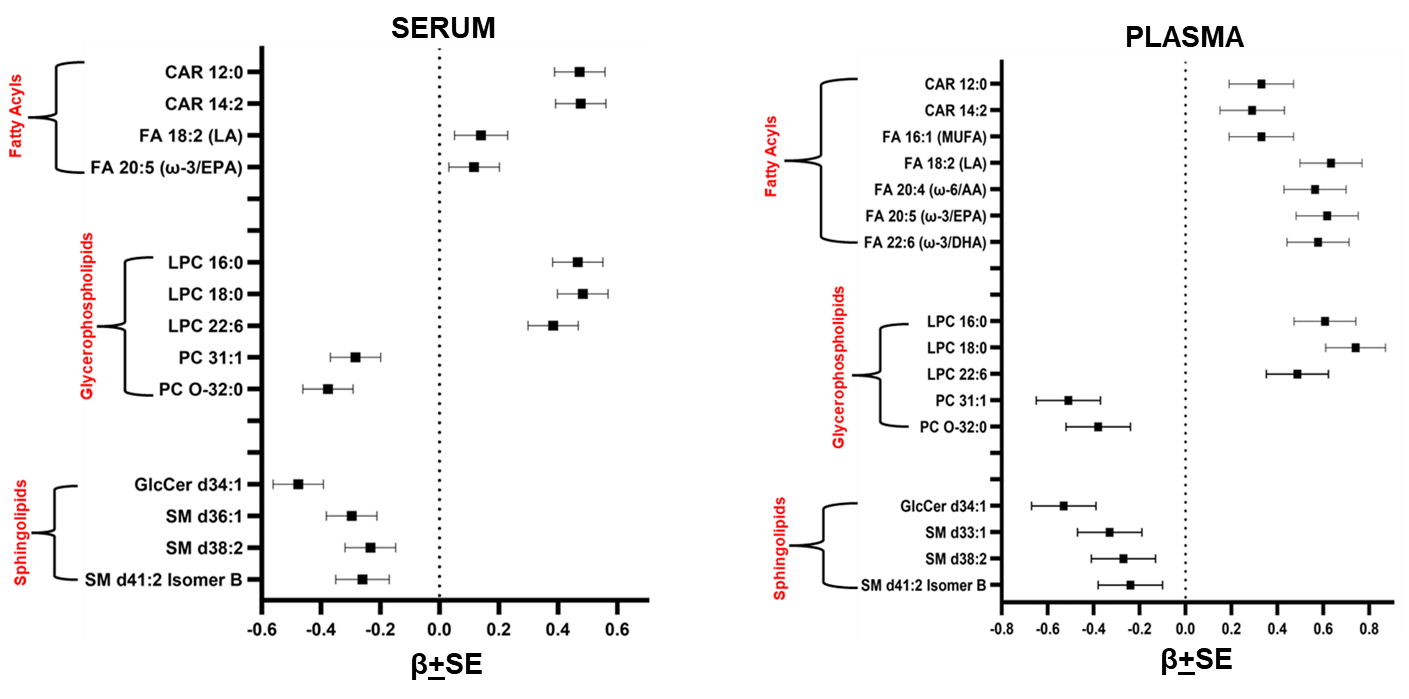


**Supplementary Figure 7**: Forest plots showing effect sizes (beta) and standard errors for clinical risk score (CRS) association with serum and plasma metabolites.


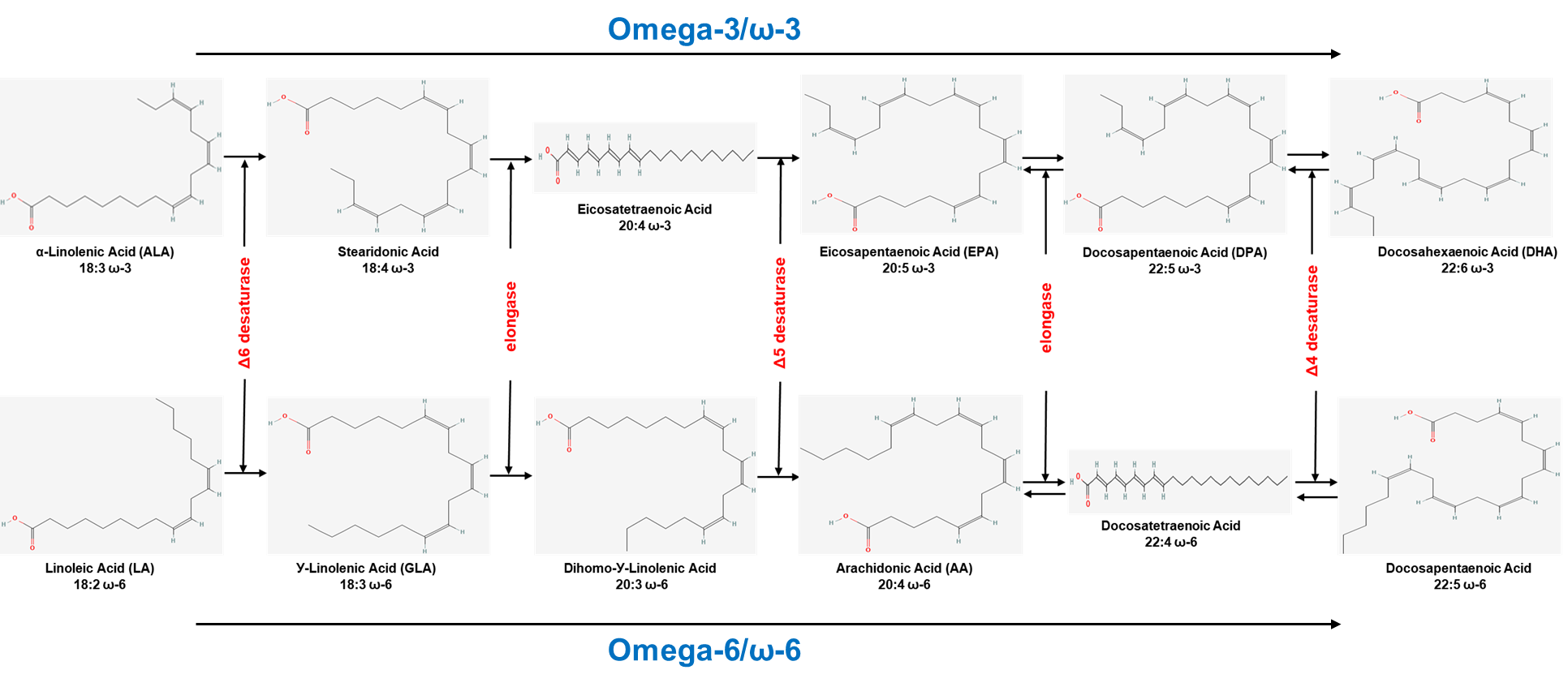


**Supplementary Figure 8**: Schematic of synthesis of ω-3 and ω-6 fatty acids
